# Bibliometric Analysis of Global Research Output on Antimicrobial Resistance among Pneumonia Pathogens (2013–2023)

**DOI:** 10.3390/antibiotics12091411

**Published:** 2023-09-06

**Authors:** Nurgul Ablakimova, Gaziza A. Smagulova, Svetlana Rachina, Aigul Z. Mussina, Afshin Zare, Nadiar M. Mussin, Asset A. Kaliyev, Reza Shirazi, Nader Tanideh, Amin Tamadon

**Affiliations:** 1 Department of Pharmacology, West Kazakhstan Marat Ospanov Medical University, Aktobe 030012, Kazakhstan; g.smagulova@zkmu.kz (G.A.S.); a.mussina@zkmu.kz (A.Z.M.); 2 Hospital Therapy Department No. 2, I.M. Sechenov First Moscow State Medical University, 119435 Moscow, Russia; rachina_s_a@staff.sechenov.ru; 3 PerciaVista R&D Co., Shiraz 73, Iran; afshinzareresearch@gmail.com (A.Z.); tanidehn@gmail.com (N.T.); amintamaddon@yahoo.com (A.T.); 4 Department of Surgery, West Kazakhstan Marat Ospanov Medical University, Aktobe 030012, Kazakhstan; nadiar_musin@zkmu.kz (N.M.M.); aset_kaliyev@mail.ru (A.A.K.); 5 Department of Anatomy, School of Medical Sciences, Biomedical & Health, UNSW Sydney, Sydney 2052, Australia; reza.shirazi@unsw.edu.au; 6 Stem Cells Technology Research Center, Shiraz University of Medical Sciences, Shiraz 71348-14336, Iran; 7 Department of Pharmacology, Medical School, Shiraz University of Medical Sciences, Shiraz 71348-14336, Iran; 8 Department for Scientific Work, West Kazakhstan Marat Ospanov Medical University, Aktobe 030012, Kazakhstan

**Keywords:** antimicrobial resistance, pneumonia, pathogens, antibiotics, bibliometrics, drug resistance, microbial, community-acquired infections, ventilator-associated pneumonia, global health

## Abstract

Antimicrobial resistance (AMR) is a pressing global concern, posing significant challenges to the effective treatment of infections, including pneumonia. This bibliometric analysis aims to investigate the research output on AMR among pneumonia pathogens from 2013 to 2023. Data were extracted from the Web of Science Core Collection (WOS-CC) using an inclusive search strategy. The analysis included 152 relevant studies published in 99 different sources, involving 988 authors and yielding an average of 16.33 citations per document over the past decade. The findings reveal a notable increase in research on AMR among pneumonia pathogens, indicating a growing awareness of this critical issue. Collaborative studies were prevalent, with the majority of authors engaging in joint research efforts. Bradford’s Law identified twelve core journals that were instrumental in disseminating research in this field, with “Medicine” emerging as the most prolific journal. The USA and China emerged as the leading contributors, while Germany displayed a strong inclination towards collaborative research. Intermountain Medical Center, Saitama Medical University, and Udice-French Research Universities were the most productive institutions, and Yayan J. and Rasche K. were the top authors. Furthermore, the analysis identified commonly encountered microorganisms such as *Acinetobacter baumanii* and *Klebsiella pneumoniae* in the context of AMR. Time-based analysis of keywords highlighted the significance of terms like “community-acquired pneumonia” and “ventilator-associated pneumonia”. Overall, this comprehensive study sheds light on the global research landscape of AMR among pneumonia pathogens. The insights gained from this analysis are essential for guiding future research priorities and collaborative efforts to combat AMR effectively and improve treatment outcomes for pneumonia and related infections. As the frequency of reports concerning resistance among pneumonia pathogens, notably *A. baumannii* and *K. pneumoniae*, continues to rise, there is an immediate requirement for pharmaceutical manufacturers and healthcare providers to respond proactively and ready themselves for the forthcoming implications of this matter. It also underscores the importance of knowledge dissemination and evidence-based interventions to address this growing public health challenge. However, the study acknowledges the limitations associated with using a single publication database and encourages the inclusion of data from other sources in future research.

## 1. Introduction

Antimicrobial resistance (AMR) is a global challenge that poses a significant risk to the effectiveness of treating a wide range of infections [1]. Furthermore, AMR also has economic implications, as it requires longer treatment durations, additional laboratory tests, and the use of costly medications [2,3]. Addressing the issue of AMR requires collective efforts from governments, healthcare providers, researchers, and the general public. Conducting research, raising awareness, enhancing knowledge, and staying updated on AMR are crucial in minimizing its impact and revising treatment guidelines for different infection types [4,5,6,7].

Lower respiratory tract infections (LRTIs) stand as the primary contributor to mortality from infectious diseases globally and hold the fifth position among all causes of death [8]. They also contribute significantly to disability-adjusted life years despite being largely preventable [9]. Over the past decade, there have been changes in the epidemiology of LRTIs, with a decrease in the burden among children under 5 years of age and an increase among individuals over 70 years old [9]. Notably, pneumococcal pneumonia accounts for 55.4% of LRTI-related deaths across all age groups [9].

The economic burden of pneumonia is substantial, as it leads to substantial healthcare costs, prolonged hospitalizations, and increased utilization of medical resources [10,11]. Moreover, pneumonia often serves as a precipitating factor for more severe conditions like sepsis, placing an immense strain on healthcare systems [12]. Understanding the role of resistant pathogens of pneumonia is crucial for both public health management and effective patient care.

Amongst the range of infections affected, pneumonia stands out as a critical area of focus, as it remains a leading cause of morbidity and mortality worldwide [13,14]. Pneumonia pathogens have increasingly developed resistance to commonly used antibiotics [15,16,17]. The misuse and overuse of antibiotics, both in healthcare and community settings, have contributed to the development of resistant strains of bacteria, rendering traditional treatment approaches less effective [18,19]. This phenomenon not only complicates the management of pneumonia cases but also diminishes the success of other medical interventions that rely on antibiotics. This alarming trend has limited treatment options and heightened the risk of severe complications [20], making it imperative to understand the evolving landscape of antibiotic resistance in pneumonia pathogens. The most significant challenges for clinicians arise when treating pneumonia caused by multidrug-resistant (MDR) pathogens [21,22].

In 2014, the World Health Organization (WHO) released the first global surveillance report on AMR to highlight the clinical consequences of resistant bacteria across WHO regions worldwide [23]. Notably, a considerable amount of time has elapsed since the publication of this initial report, and it is important to acknowledge the transformative impact of the COVID-19 (Coronavirus disease 2019) pandemic, during which there has been an elevated utilization of antibiotics, especially for the patients with pneumonia [24]. Therefore, analyzing the published literature on AMR is crucial for the development of new empirical and therapeutic guidelines for the treatment of pneumonia. Additionally, examining the published literature is essential for understanding the global and regional changes in AMR. While several review articles have discussed AMR among pathogens in the context of LRTIs [25,26,27,28], none have specifically analyzed the global research output and trends in this area. Bibliometric analysis proves to be a powerful approach in quantitatively evaluating academic papers, enabling researchers to explore the progression of specific fields; it is a valuable tool that employs mathematical and statistical methods to assess the growth, productivity, and overall patterns of publications related to a specific topic [29,30]. In the medical field, such analyses play a crucial role in illustrating research trends and developments, motivating researchers to identify leading countries and institutions, as well as areas that require improvement. This study will serve as a foundational source of information for future comparisons and assessments. It aims to identify trends and advancements in this area of research and shed light on potential areas for further focus and improvement.

## 2. Results

### 2.1. Summary of the Papers

This study aimed to comprehensively examine the global research output on AMR among pathogens associated with pneumonia, covering articles published from 2013 to 2023. A total of 152 relevant studies were carefully reviewed, originating from 99 different sources. The analysis involved the contributions of 988 authors, collectively producing an impressive average of 16.33 citations per document over the past decade. The key findings related to antimicrobial resistance in pneumonia pathogens are presented based on the highest-cited documents over the past decade in Table 1. In addition, the Annual Growth Rate for this research field was calculated to be 2.03%, indicating a steady increase in publications over the study period. The vast volume of research output is further highlighted by the inclusion of 4351 references and 394 unique author keywords. The overwhelming majority of authors participated in collaborative studies (98%).

### 2.2. Trend of Publication and Citation

**Figure 1:**
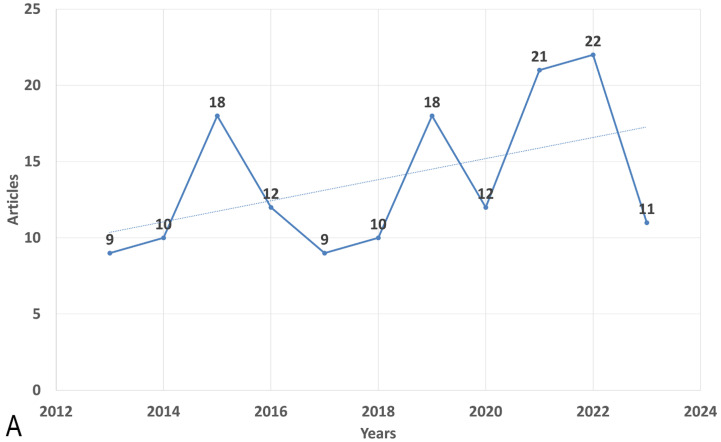

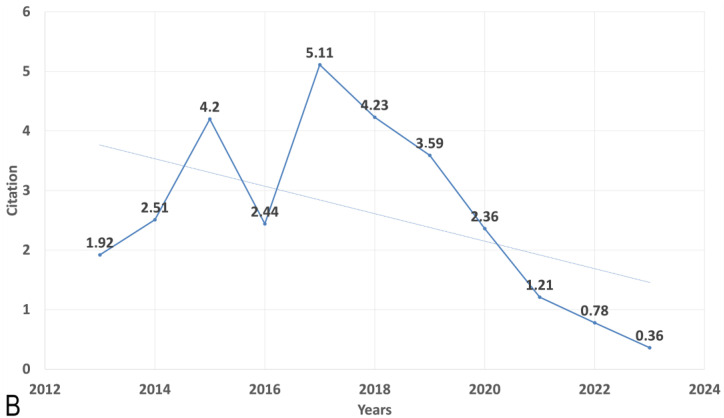
Global annual trend of (**A**) publication and (**B**) citation on the antimicrobial resistance among pneumonia pathogens (2013–2023). Dotted lines show the trendlines for better demonstration of trend of increase of articles number and trend of decrease of citations during the period of time.

The data indicate fluctuations in the mean total citations per article across the years. In 2015, there was a substantial increase in the mean total citations per article, reaching 37.83, while 2023 recorded the lowest value of 0.36. Furthermore, the number of publications (N) varied from year to year, with the highest number of articles published in 2022 (N = 22) and the lowest in 2013 (N = 9). Figure 1 illustrates the publication trend over the years (Figure 1A).

**Figure 2:**
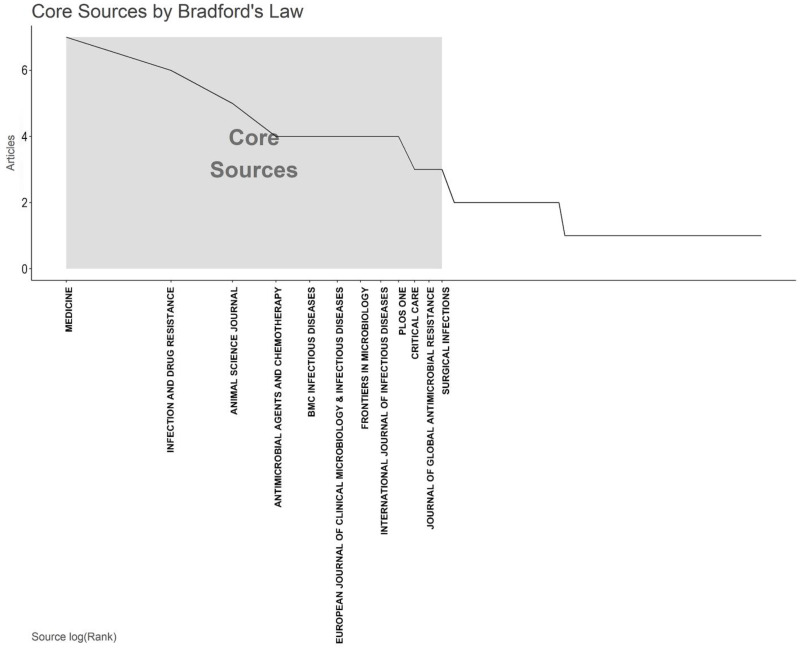
The plot of Broadford’s Law identified twelve core journals on the antimicrobial resistance among pneumonia pathogens (2013–2023).

Utilizing Bradford’s Law, which describes the distribution of scientific articles among different journals, we identified twelve core journals that were deemed to be the top choices for researchers (Figure 2). According to Bradford’s Law, these core journals collectively accounted for a significant portion of the total number of articles published on AMR among pneumonia pathogens. Upon analyzing the publication data from these core journals, we observed that the journal “Medicine” emerged as the most prolific one, contributing a substantial seven articles, which represents approximately 4.6% of the total articles within the study period. Furthermore, we investigated the local citations received by these core journals from other articles within our dataset. Remarkably, “Clinical Infectious Diseases” stood out with the highest number of local citations, amassing an impressive total of 238 citations (Table 2).

### 2.3. Most Productive Authors, Institutions, Countries and Their Collaboration Network

**Figure 3:**
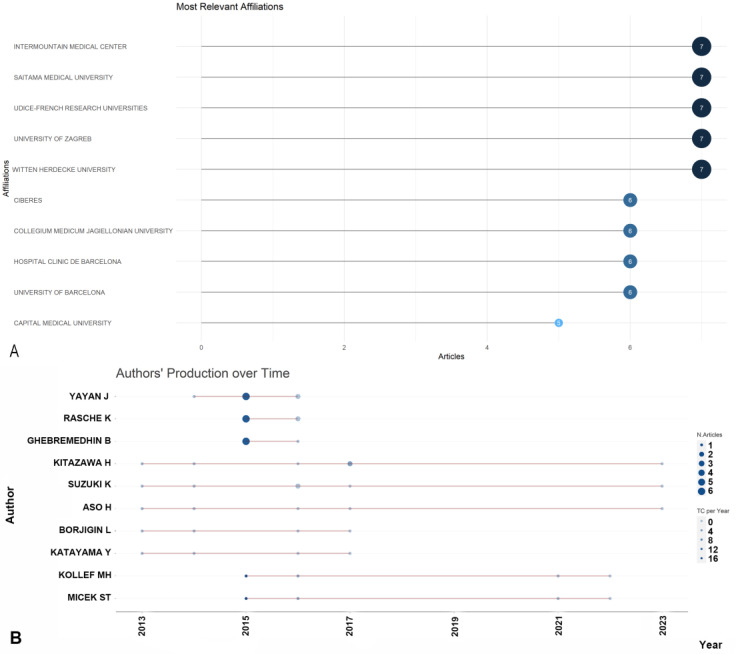
Most productive authors, institutions, countries and their collaboration network (**A**). (**B**) Ten most-contributing authors and their production over time on the antimicrobial resistance among pneumonia pathogens (2013–2023).

**Figure 4:**
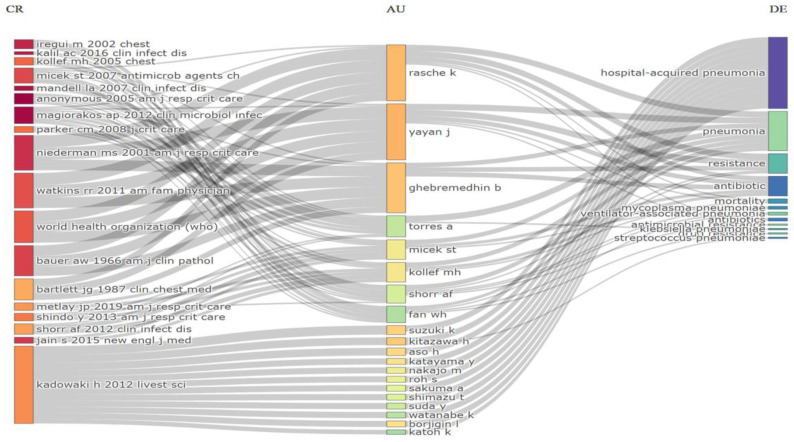
Three-Fields Plot representing the incoming and outgoing flows among cited references, authors and author keywords contributing to antibiotic resistance among pneumonia pathogens (2013–2023). Abbreviations: CR, cited references; AU, authors; and DE; keywords. Explanations of the right columns (DE) from top to bottom: hospital acquired-pneumonia; pneumonia; resistance; antibiotics; mortality; *Mycoplasma pneumoniae*; ventilator-associated pneumonia; antibiotics; antimicrobial resistance; Klebsiella pneumoniae; drug resistance; *Streptococcus pneumoniae*.

Intermountain Medical Center (Murray, UT, USA), Saitama Medical University (Moroyama, Japan), Udice-French Research Universities (Paris, French), University of Zagreb (Zagreb, Croatia), Witten Herdecke University (Witten, Germany) emerged as the most productive institutions, contributing the highest number of articles (7, 4.6%) (Figure 3A). Among the authors, Yayan J. stood out with the highest number of articles (9, 5.9%), followed by Rasche K., who produced eight articles (Figure 3B). The Three-Fields Plot vividly illustrates the intricate web of connections among cited references, authors, and author keywords, providing invaluable insights into the complex landscape of antibiotic resistance among pneumonia pathogens during the decade spanning from 2013 to 2023. (Figure 4).

**Figure 5:**
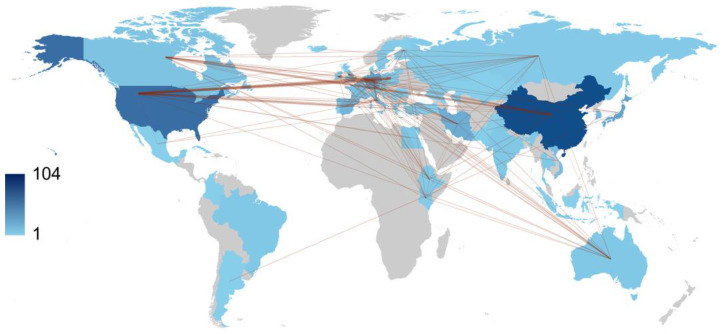
World collaboration map on the antimicrobial resistance among pneumonia pathogens (2013–2023). The intensity of color saturation corresponds to the increasing number of articles within each country. The collaboration between countries is depicted through the thickness of the connecting arrows.

Over the course of one decade, China and the USA led the scientific production among countries with 104 and 101 publications, respectively, followed by Japan with 50 publications, Spain with 32, and Vietnam with 30 (Table 3). Regarding publication patterns, China exhibited a significant tendency for single-country productions, with 83.7% of its publications. Similarly, the United States demonstrated a high rate of single-country publications at 82.2%. In contrast, Germany engaged in collaborative research, with 65.4% of German authors’ publications being co-authored with researchers from other countries. Collaboration strength was mostly derived from the United States and European countries (Figure 5).

### 2.4. Co-Occurrence, Hotspots and Emerging Keywords

**Figure 6:**
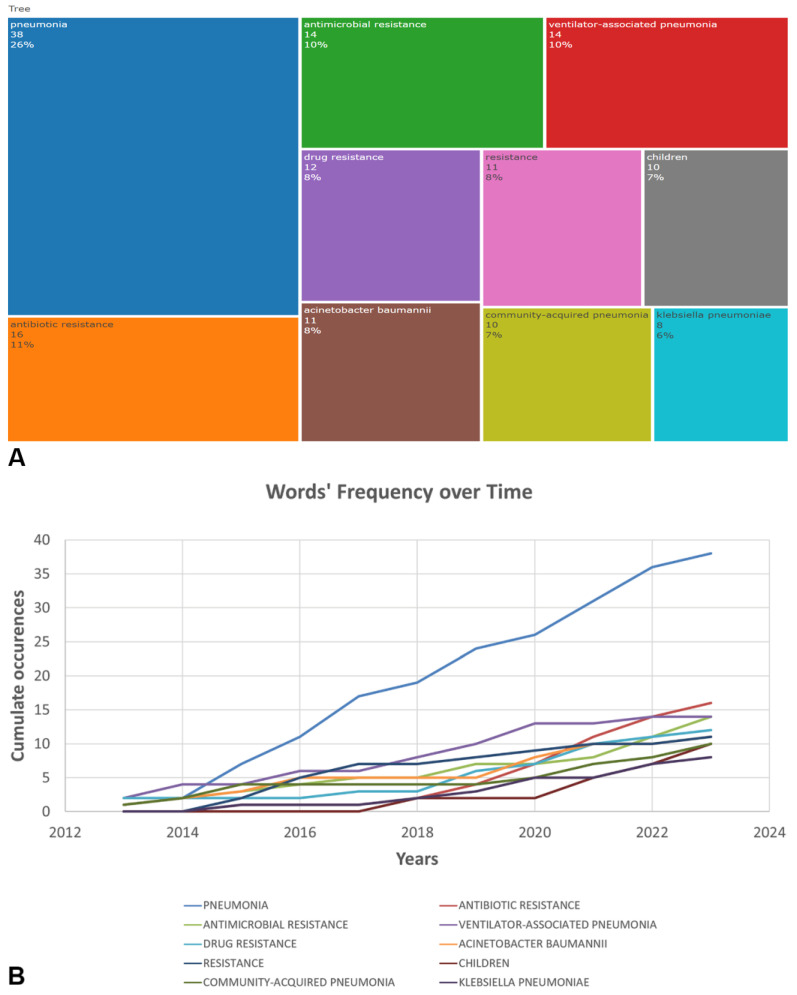
TreeMap (**A**) and scatter plot (**B**) representing top ten author’s keywords in the research on the antimicrobial resistance among pneumonia pathogens (2013–2023).

The most commonly found author keywords were examined using Biblioshiny. The analysis covered commonly encountered antimicrobial agents, microorganisms, types of pneumonia and terms related to AMR. Keywords related to antibiotic resistance (“antibiotic resistance”) and antimicrobial resistance (“antimicrobial resistance”) both exhibited an upward trend, with 16 and 14 occurrences in 2023, respectively. The frequency of the keyword “ventilator-associated pneumonia” remained relatively stable over the years, with 14 occurrences in 2023. Research on *Acinetobacter baumannii* showed a notable increase, with 11 occurrences in 2023, indicating growing attention to this MDR pathogen. Keywords related to *Klebsiella pneumoniae* demonstrated steady research interest over the years, with eight occurrences in 2023, reflecting continued efforts to address this problematic pathogen (Figure 6).

**Figure 7:**
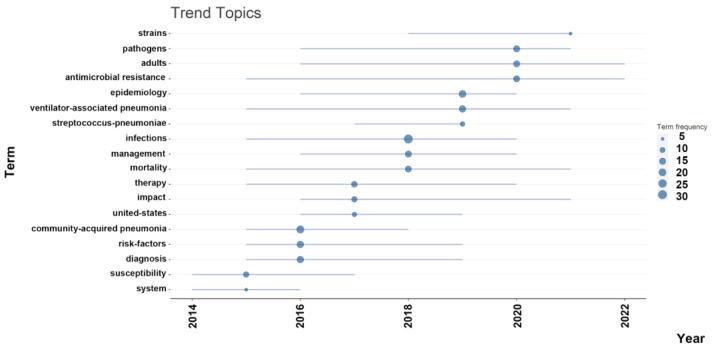
The timeline of the trend topics. Each bubble indicates the peak of frequency used for each, while the line indicates the years it was used.

The timeline analysis of important keywords reveals that “community-acquired pneumonia” received peak citations in 2016, with “ventilator associated pneumonia” remaining relevant during the pandemic. Topics such as Epidemiology (19%), Diagnosis (17%), and Risk Factors (17%) also have considerable frequencies, demonstrating the sustained interest in understanding the epidemiology, diagnosis, and risk factors of bacterial pneumonia infections (Figure 7).

In summary, this study comprehensively reviewed global research output on AMR among pneumonia pathogens over the past decade, identifying top journals, impactful articles, collaborations between institutions, authors, and countries, as well as important and emerging keywords. The findings provide valuable insights into the research landscape and highlight potential areas for future studies.

## 3. Discussion

In recent years, bibliometric analysis and scientific mapping have been rapidly advancing because the scientific community has shown a growing interest in the outcomes of various bibliometric analyses [41]. Bibliometric analysis allows for exploring the impact of research fields, the influence of researchers, the significance of individual papers, and can help identify particularly influential papers, thereby enhancing our understanding of the overall intellectual landscape within specific research fields [42].

The analysis revealed a notable increase in research output on AMR among pneumonia pathogens over the past decade [22,43]. This growth indicates a growing awareness of the critical issue of AMR and its impact on the effective treatment of pneumonia. According to Cillinoz et al., the incidence of MDR Gram-negative bacteria is rising among cases of nosocomial pneumonia, such that it is now becoming a significant challenge for clinicians [43]. The increasing number of publications reflects the urgency and importance of addressing this global health challenge, particularly in the context of utilizing reserve antibiotics and exploring novel combinations to combat it effectively [22,44,45].

The analysis of global research output on AMR among pneumonia pathogens over the past decade reveals interesting trends and insights. One noticeable shift in research focus is the transition from community-acquired pneumonia (CAP) to hospital ventilator-associated pneumonia (VAP). VAP has gained prominence as a critical concern due to its association with prolonged hospital stays [46], increased morbidity and mortality rates [47], and the higher likelihood of drug-resistant pathogens [45]. Researchers and healthcare professionals have directed their attention to understanding the underlying mechanisms of AMR in VAP to develop effective treatment strategies and infection control measures. The high interest in VAP may be associated with COVID-19 because nearly half of patients with COVID-19 admitted to ICU may develop VAP, with a pooled estimate of mortality of 42.7% for COVID-19 patients who developed VAP [48]. The high 30-day case fatality of VAP likely represents the sum of the prognostic effects of the underlying viral and superimposed bacterial diseases [49]. This shift may be attributed to the increasing recognition of nosocomial infections and the significance of AMR in healthcare settings.

One of the most frequently cited studies addresses the issue of *Staphylococcus aureus* resistance, and the research was conducted using an animal model [34]. This underscores the significance of such studies in exploring diseases caused by resistant pathogens. Gaugaet et al. emphasized that further studies are needed to examine the link between the gut microbiota and the production of certain antimicrobial proteins in the lung. The potential use of gut microbiota as an alternative to antibiotics is an emerging area of research and interest in the medical field [50,51]. The concept revolves around harnessing the natural antimicrobial properties of the gut microbiota to combat infections without resorting to traditional antibiotic treatments. This approach has gained attention due to concerns about antibiotic resistance.

Aguilar et al. [52] estimated that in 2019, AMR was linked to approximately 569,000 deaths (95% UI 406,000–771,000) and about 141,000 deaths (99,900–196,000) were connected to bacterial AMR in the 35 countries within the World Health Organization’s Region of the Americas. The six leading pathogens (by order of number of deaths associated with resistance) are *S. aureus*, *Escherichia coli*, *K. pneumoniae*, *Streptococcus pneumoniae*, *Pseudomonas aeruginosa*, and *A. baumannii*. Together, these pathogens were responsible for 452,000 deaths (326,000–608,000) associated with AMR.

Our bibliometric study incorporated research related to all the aforementioned pathogens. Between 2017 and 2019, *S. pneumoniae* was identified as one of the trending topics. Reports of multidrug-resistant strains of *S. pneumoniae* have been published since the late 1970s [53]. The global rise in antibacterial resistance within pneumococci continues, particularly affecting β-lactams and macrolides [54,55]. In nations like France and Spain in Europe, up to 40% of pneumococcal strains exhibit resistance to multiple drugs [56].

However, the analysis of author keywords highlights the prominence of *A. baumannii* and *K. pneumoniae* among commonly encountered microorganisms in the context of AMR and pneumonia. Both of these pathogens belong to the ESKAPE group, which includes bacteria known for their high resistance to multiple antimicrobial agents [57,58].

Researchers observed a wide variation in the prevalence of MDR among *A. baumannii* causing HAP and VAP (ranging from 55% to 100%) and mortality rates (ranging from 28% to 68%) between regions and countries [15]. Based on previous research, the primary risk factors for VAP caused by *A. baumannii* include antibiotic therapy, especially broad-spectrum antibiotics, mechanical ventilation, prolonged hospital stay, invasive procedures, disease severity, and the presence of chronic diseases [59,60]. In a study by Inchai et al. [61], carbapenem treatment was associated with the risk of VAP caused by all three types of *A. baumannii* drug-resistant profiles, particularly pandrug-resistant (PDR), with odds ratios of 5.2, 6.3, and 12.84 for MDR, extensively drug-resistant (XDR), and PDR *A. baumannii* VAP, respectively. The use of carbapenems was also identified as a risk factor for XDR *A. baumannii* VAP in the study by Li et al. [60]. Additionally, previous colistin treatment was found to increase the likelihood of VAP caused by PDR *A. baumannii* [61].

The current mechanism of resistance of *K. pneumoniae* involves the production of enzymes called beta-lactamases, which include extended-spectrum ß-lactamases, cephalosporinases, and carbapenemases [62,63,64]. The presence of carbapenemases and extended-spectrum beta-lactamases in nosocomial *K. pneumoniae* has resulted in a MDR phenotype, significantly limiting available treatment options [62].

The higher prevalence of AMR in *A. baumannii* and *K. pneumoniae* underscores the urgency of targeted research efforts to combat drug resistance in these pathogens. These findings align with the global concern over the rapid spread of drug-resistant infections, emphasizing the need for effective surveillance, infection prevention measures, and novel antimicrobial therapies.

Our study is the first study to access AMR among pneumonia pathogens in a bibliometric way. However, it is essential to acknowledge the limitations of this study, which are associated with the use of a single publication database. WOS has covered many publications; however, some publications from databases, such as Scopus and PubMed, may not be included in this study. Due to our bibliometric analysis approach using empirical data from original articles, we focused on article metadata rather than content, extracting author details, institutions, and countries for productivity, collaboration, and impact assessment. Textual content analysis was not included. Furthermore, it should be noted that only English-language full-text articles were analyzed. Even if abstracts were available in English, such publications were excluded. As a result, certain articles from journals published in national languages might not have been included in the analysis.

## 4. Materials and Methods

### 4.1. Search Results

In July 2023, data were collected from the Web of Science Core Collection (WOS-CC) to comprehensively analyze research on AMR among pneumonia pathogens. The search strategy used in this study aimed to be inclusive and covered various aspects of the topic. The search strategy employed was as follows: “resistance” AND “pneumonia” (Title) and “resistance” AND “pneumonia” (Abstract).

**Figure 8:**
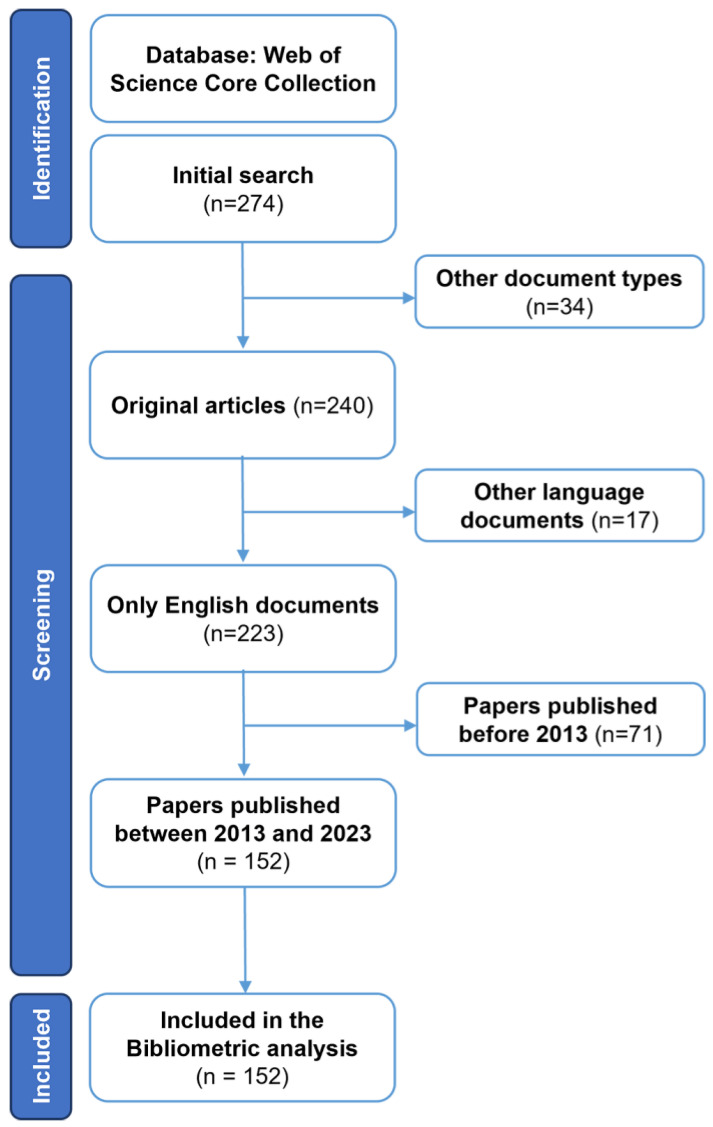
The flow chart of the screening process using PRISMA.

To ensure the accuracy and relevance of the data, specific inclusion criteria were applied. These criteria included: (1) articles published between 2013 and 2023, (2) articles written in English, and (3) exclusion of review articles, proceeding papers, book chapters and editorial material. To provide a visual representation of the data extraction process, a PRISMA flowchart outlining the selection process is presented in Figure 8.

### 4.2. Performance Analysis

In this study, performance analysis and science mapping were conducted using specific software tools. Rstudio v.4.3.1 with the bibliometric R-package (http://www.bibliometrix.org; access date: 19 July 2023), was utilized for these analyses [65]. Biblioshiny, with its web features, was employed for data analysis. This software has the capability to function with a single database only. WOS was chosen due to its provision of comprehensive and detailed citation information. This feature is particularly valuable for conducting thorough bibliometric analysis and assessing the impact of research outputs.

The local publication trends and average total citations per article were measured for each year. To identify the most productive journals, the number of publications was considered, and Bradford’s Law was applied to identify core journals, which are a few journals contributing significantly to citations in the field [66].

### 4.3. Identification of Leading Institutions, Sources, Authors, and Collaborating Countries

The top 10 most productive institutions and authors were ranked based on the percentage of papers they produced. Relationships between institutions and authors were visualized to understand their collaboration patterns. For country analysis, the percentage of articles from each country was used to rank the most productive country, and the percentage of multiple-country production was measured for the top 10 countries. The country collaboration network was mapped based on the number of publications produced by each country.

### 4.4. Keywords Frequencies Analysis

Timeline analysis was performed to observe how frequently specific keywords appeared over the years. TreeMap was created to illustrate the distribution and prominence of the top 10 most frequently occurring keywords. Thematic analysis was conducted to identify the main trends and topics within the selected articles.

## 5. Conclusions

Over the past decade, research on AMR among pneumonia pathogens has shown a significant increase. China and the USA have made significant contributions to this field, as evidenced by their representation among the authors of 104 and 101 articles, respectively. Antibiotic-resistant bacterial infections in Africa are still of great concern as they remain poorly understood. It is crucial to update and share research findings from different countries regarding AMR with clinicians to develop appropriate empirical therapy for pneumonia. Addressing this growing health challenge requires converting global data on AMR among pneumonia pathogens into a comprehensive action plan. With an escalating number of reports on resistance among pneumonia pathogens, especially *A. baumannii* and *K. pneumoniae*, there is an urgent need for pharmaceutical companies and healthcare providers to take action and prepare for the future consequences of this issue.

## Figures and Tables

**Table 1:** The top 10 most cited documents on antimicrobial resistance among pneumonia pathogens (2013–2023).

Rank	Study ID [References]	Title of the Document	Journal Name	Total Citations	DOI
1	Shields RK, 2018 [31]	Pneumonia and renal replacement therapy are risk factors for ceftazidime-avibactam treatment failures and resistance among patients with carbapenem-resistant Enterobacteriaceae infections	*Antimicrobial Agents and Chemotherapy*	174	10.1128/AAC.02497-17
2	Micek ST, 2015 [32]	An international multicenter retrospective study of Pseudomonas aeruginosa nosocomial pneumonia: impact of multidrug resistance	*Critical Care*	154	10.1186/s13054-015-0926-5
3	Zilberberg MD, 2017 [33]	Carbapenem resistance, inappropriate empiric treatment and outcomes among patients hospitalized with Enterobacteriaceae urinary tract infection, pneumonia and sepsis	*BMC Infectious Diseases*	131	10.1186/s12879-017-2383-z
4	Gauguet S, 2015 [34]	Intestinal Microbiota of Mice Influences Resistance to Staphylococcus aureus Pneumonia	*Infection and Immunity*	127	10.1128/IAI.00037-15
5	Yayan J, 2015 [35]	Antibiotic resistance of Pseudomonas aeruginosa in pneumonia at a single university hospital center in Germany over a 10-year period	*PLoS ONE*	105	10.1371/journal.pone.0139836
6	Martin-Loeches I, 2015 [36]	Resistance patterns and outcomes in intensive care unit (ICU)-acquired pneumonia. Validation of European Centre for Disease Prevention and Control (ECDC) and the Centers for Disease Control and Prevention (CDC) classification of multidrug resistant organisms	*Journal of Infection*	105	10.1016/j.jinf.2014.10.004
7	Lee SH, 2019 [37]	Performance of a multiplex PCR pneumonia panel for the identification of respiratory pathogens and the main determinants of resistance from the lower respiratory tract specimens of adult patients in intensive care units	*Journal of Microbiology, Immunology and Infection*	87	10.1016/j.jmii.2019.10.009
8	Zilberberg MD, 2016 [38]	Multidrug resistance, inappropriate empiric therapy, and hospital mortality in Acinetobacter baumannii pneumonia and sepsis	*Critical Care*	76	10.1186/s13054-016-1392-4
9	Fernandez-Barat L, 2017 [39]	Intensive care unit-acquired pneumonia due to Pseudomonas aeruginosa with and without multidrug resistance	*Journal of Infection*	65	10.1016/j.jinf.2016.11.008
10	Jamal W, 2014 [40]	Evaluation of Curetis Unyvero, a multiplex PCR-based testing system, for rapid detection of bacteria and antibiotic resistance and impact of the assay on management of severe nosocomial pneumonia	*Journal of Clinical Microbiology*	59	10.1128/JCM.00325-14

**Table 2:** The top 10 most cited journals on antimicrobial resistance among pneumonia pathogens (2013–2023).

Sources	Articles
*Clinical Infectious Diseases*	238
*Antimicrobial Agents and Chemotherapy*	227
*Journal of Clinical Microbiology*	114
*American Journal of Respiratory and Critical Care Medicine*	110
*Journal of Antimicrobial Chemotherapy*	110
*Clinical Microbiology and Infection*	100
*PLOS ONE*	92
*Chest*	90
*Critical Care Medicine*	81

**Table 3:** Leading publishing countries on antimicrobial resistance among pneumonia pathogens (2013–2023).

Country	Number of Articles
China	104
USA	101
Japan	50
Spain	32
Vietnam	30
Iran	27
Germany	26
UK	24
South Korea	19
India	13

## Data Availability

Data are contained within the article.
